# Food—Animal and Vegetable

**Published:** 1884-12

**Authors:** E. J. Lilly

**Affiliations:** Circleville, O.


### Food—Animal and Vegetable.

BY E. J. LILLY, M.D., D.D.S., CIRCLEVILLE, O.

Read before the Ohio State Dental Society.

A food, according to the usual definition, is a substance which, when introduced into the body, supplies material to renew some structure, or maintain some vital process. And most people show, in every-day conversation, that they consider the main object of food to be the replacement of the *materials* of the organism, while, in fact, its real object is the renewal of the *energies* which have been dissipated in automatic and voluntary activities, or, in other words, work. A man, like a steam engine, cannot produce motion or give out energy, without his appropriate fuel, and, if the fuel is not supplied, the fire goes out and the man dies.

Food-stuffs and coal agree essentially in the chief characteristics of their chemical composition. Both consist mainly of hydrogen and carbon, and both possess energy in virtue of the fact that their affinities for oxygen are not satisfied. Water contains hydrogen, and carbonic acid contains carbon; but we can get no energy out of these, because in them the oxygen has already united with the atoms for which it had affinity, and the separation necessary for dormant energy has ceased to exist. But in bread, potatoes, meat, or coal, the hydrogen and carbon are ready to unite with their equivalent of oxygen whenever the chance presents. All alike obtained their energy in the same way. The rays of sunlight falling upon their original trees or plants, seperated the oxygen, from the water, and carbonic acid gas in the air, and built up the hydrocarbons in their tissues. The force thus stored up remains dormant until an opportunity occurs to reunite with oxygen, when it will once more assume the active shape; producing mental, physical, and mechanical energy, as needed. The only serious difference between food and coal is that foods contain another element, nitrogen, as well as hydrogen and carbon, and this nitrogen is absolutely necessary, if the animal is to continue to live.

Foods are derived from earth, water, and air, and may be solid liquid, or gaseous, organic and inorganic. The popular notion of food as solid substances derived from animals and vegetables, while comprehensive, is too exclusive, since the water we drink, the air we breathe, and certain mineral substances found in the earth, are of no less importance as foods. It is understood that the structures of the body are in a state of continual change, so that atoms and molecules which are present at one hour may be gone the next; and when thus used up by work, the tissues will be so far wasted, unless the process of waste be accompanied by renewal. This is brought about by the ingestion of such materials as, by vital activity, may be transformed into tissue.

Vegetable food contains, as its essential nutritive elements, albuminates or nitrogenous compounds, fatty matter, and hydrates of carbon, with water and nutritive salts. Such food, therefore, contains the same elements as that derived from animals ; but in point of digestibility the two differ widely, meat being very much more easily digested than vegetable food. What renders the latter harder to digest is the fact that the albuminoid substances, fatty matters, and starch are there incased in a coating of cellulose, to break up which requires some time. Starch, before it can be absorbed, must first be changed into sugar, which process may occur in the mouth, if the food be chewed long enough, or in the intestines, when it comes in contact with the pancreatic and intestinal juices.

Of all the influences which determine the life of the individual, and on which his weal and woe depend, undoubtedly the nature of his food is one of the weightiest. Every one has for himself experienced how not only the strength of his muscles, but also the course of his thought and whole mental tone are affected by the nature of his food, whether it be strong and wholesome, or otherwise. Every nation has its peculiar way of cooking; but the Yankee mode of spoiling eggs, and concocting rank and indigestible dishes, delusive to the eye, deceptive to the palate, and a deadly snare to the digestive organs, is purely American. The stomach may not be the seat of the soul, but the road to the heart and soul, and the health of the brain, lies through it; and, indeed, it is said that " a man's heart and stomach are interchangeable terms."

Foods have been classed into different groups, according to the influence they have on the body, in virtue of their essential constituents. And though the classification, like every artificial arrangement in nature, is only approximately correct, still it gives some ground to stand on. Blood-formers, or albuminates, are those albuminous materials which constitute the nutritive elements of the blood, and enter into the composition of the ligaments, bones and muscles, on which the exertion of force specially depends. The heat-producers, or respiratory foods, are those rich in carbon ; these specially serve to support, with the aid of inspired oxygen, the process of combustion so necessary for the purposes of the organism. Finally, there is a third group of substances—the nutritive salts—which are of an inorganic nature, and which, after the combustion of the food remain in the shape af ash. All these food materials are essential, since with them the organism is built up. Life is an unceasing process of waste and repair, and the food must make good the loss the tissues suffer every instant. Even those substances which are contained in the living body only in small quantities must be supplied, for on these depends the activity of important organs. Such substances are common salt, magnesia, lime, phosphorous, iron, and others less common. But none of these groups is, by itself, sufficient for nutrition. They must all be combined. Blood-formers, heat-producers, or nutritive salts are not separately foods, but only factors of food; each as indispensable to the organic processes as air and water, but each incapable by itself of supporting life. One cannot live on albumen alone, or fat alone Without the phosphate* and other salts of lime neither bone nor tooth substance would be formed, no matter how much pure albumen we ate; on the other hand, without albumen no muscular tissue would be made, though we were to gorge ourselves with sugar and salt. Finally, without fat, no brain. But we, properly enough, give the name of food to milk, meat, and bread, for in them all the three conditions are present, but in different proportions; and, in order to obtain them in certain definite quantaties, mixed foods must be used. Now, in nature they are not distributed in any such definite proportions, there the greatest diversity is found. One food stuff consists principally of blood-formers; another of heat-producers; this one contains only one of the nutritive salts, that one another. However, we must not subsist on a too mixed diet; for if one swallows without thinking, and often without knowing, incompatible and warring articles of food or refreshments at the same meal, he turns his stomach into a sort of chemical laboratory, or fermenting tank. Such experiments are sure to make disturbance and various internal commotions, disagreeable and perilous in their very nature. It should need no chemical analysis to tell us this, experience is enough.

---

*Not even the primary cells, without the phosphate.—WATT.

There are some who make wheaten food, and not beef, the basis of alimentation. Wheat and the allied sub-foods, including beans, lentils, peas, and rice, taking the place of animal foods, including besides fleshmeats, butter, cheese, eggs, and milk. Sound, ripe wheat, deprived of its outer silicious husk, coarsely ground and mixed with water, and subjected to just that degree of kneading and baking which will suffice to prepare it for mastication and the subsequent processes, is a substance that must be chewed, as it cannot be swallowed without due mastication and insalivation, and consequently its digestion is assured. Wheat, coarsely ground and unbolted, contains all the natural nutritive elements of the grain. The salts of lime, in unbolted flour, are instrumental in the production of firm, strong bones and teeth. If this is excluded the bread is no better in any way. It is estimated that every child consumes one half barrel of flour every year. If this is true, and it is fed on fine white flour, it is yearly deprived of about twenty pounds of the elements that ought to be taken into the system to make solid bones and teeth. Besides, this coarsely ground grain possesses the mechanical properties which distend the intestines, promoting their peristaltic action. It is, therefore, anti-dotal to dyspepsia. For children it is specially valuable, and its substitution for common bread, with the use of fruits instead of flesh, until the deciduous teeth shall have given place to the permanent denture, would be of incalculable value and benefit, and would contribute not a little to the formation of perfect teeth. This process of feeding might be kept up throughout childhood, and even adult life, for, as stated above, in vegetables we have foods closely analagous to the flesh of animals. Thus, in addition to the water and salts, common to both, there is vegetable jelly, vegetable albumen, vegetable fibrine and caseine, all having a composition almost identical with animal albumen, gelatine, chondrine, and caseine. Such vegetable foods as contain albumen, when taken with those containing much starch, notably aid in the digestion of the latter. And still, taking an equal quantity of vegetable albumen and animal albumen, twice as much of the latter will be absorbed. This difference in absorption makes the essential difference between the two kinds of food. Bread, rice, potatoes, maize, taken in any quantity whatsoever, can scarcely support the life of any man or carnivorous animal, communicating to them no bodily strength. Too large a proportion of their nutritive elements is eliminated in the excretion. Yet, with the addition of a small quantity of albumen, they may suffice, and this can be obtained in no better or easier manner than by taking some meat food. Of course one may sustain life on purely and distinctively vegetable fare. The vegetable eater, pure and simple, can extract from his food all the principles necessary for life and its activities, provided he selects vegetables which contain these elements. But he must consume the best cereals, wheat or oats, or the legumes, peas, beans, or lentils, or else he must swallow and digest a large weight of vegetable matter of less nutritive value, and therefore, containing one element in great excess, in order to obtain what he needs. All this waste of digestive energy could be saved by the judicious admixture of animal and vegetable materials. So the question as to whether man is designed to be a vegetable feeder, a flesh eater, or an omnivorous animal, seems to favor the latter supposition. His teeth show that he is and has been omnivorous, to the extent of his means. And as man is physiologically constituted so as to be able to derive all that is necessary to the healthy performance of his functions from the animal or from the vegetable kingdom, either singly or combined, he can scarcely be regarded otherwise than as qualified to be omnivorous. Add to this his possession of an intelligence, which enables him to obtain food of all kinds from all climes, to investigate its qualities and render it more fit for digestion, by the use of heat and of condiments—powers which no other animal possesses—and there appears no *a priori* reason for limiting his diet to the products of either kingdom exclusively. Everyone likes to find his food agreeably flavored, and we have it prepared in such a way as to acquire the peculiar flavor that pleases us. For the same reason we like a variety in food. In time the persistent impression of one flavor produces disgust, just as the continued use of one article of food will cause a dislike for it, after awhile, and so what was once savory now becomes unpleasant.

A man who is chopping wood, in an atmosphere at zero, and he who uses only his brain, in a room at seventy degrees, consume very different elements in very different proportions, and, therefore require very different elements of food. In the tropics, of course, the most wholesome and agreeable diet is vegetable in its nature; in temperate regions this is supplemented by milk, (butter and cheese,) poultry, fish and different meats; while in colder regions, vegetable products are hardly to be obtained, and flesh and fat are indispensable. Thus it appears that *man* is clearly omnivorous, while *men* may advantageously be vegetarian in one climate, mixed feeders in another, and exclusively flesh-eaters in a third. For all who are occupied with severe and continuous mechanical labor, a mixed diet, of which cereals and legumes form a large portion, and meat, fish, eggs, and milk a moderate proportion, is more nutritious than chiefly animal food. For those whose labor is mental, and whose muscular exercise is inconsiderable, still less of concentrated nitrogenous food is desirable. A liberal supply of cereals and legumes, with fish and flesh in their lighter forms, will better sustain such activity than large portions of butcher's meat, two or three times a day. But after all, the old saying that "We live, not by what we eat, but by what we digest," is true; and what one man may readily digest another will die in attempting.

---

PITTSBURGH, PA., Oct. 21, 1884.

*Dr. Berry, President Ohio State Dental Society:*

DEAR FRIEND—Your letter requesting my presence, or a paper, at the coming meeting of your State Society, was received in due time. You will please excuse the long delay of my reply; and as I now respond, I do so saying that I feel compelled to deny myself, this year, the pleasure it always gives me to meet with your State Association. Having been benefitted by your meetings in the past, it would seem but simply just for me to make some effort to give something in return, but I must ask to be excused on the ground of incompetency, though I would like to make one or two suggestions; first in behalf of *Prosthetic or*

*Artificial alias Mechanical Dentistry*, which seems to be beneath the dignity of many who sport the title of D.D.S., (and we fear above the abilities of most of those who so regard that portion of our avocation). As the operative department of our calling has advanced during the past twenty years, the artificial has correspondingly retrograded. While one has progressed, with wondrous rapidity, not in one thing or in one particular line merely, but in everything pertaining to that branch of special surgery, until instruments, appliances, and methods have so multiplied, until it seems that the operative dentist has much more to learn than the general surgeon. To all advancement we bid a cordial welcome. Though all such improvements tend towards, yet they will never obviate, the necessity for artificial dentures in many cases.

While this has been doing, artificial dentistry has been neglected, and allowed to drift into the hands of the incompetent and ignorant, whose limited knowledge of the business consists, merely in the manipulation required to produce a so-called set of artificial teeth, on a rubber, or a still greater humbug, a celluloid base. Their knowledge of routine work has cost them nothing; and having no profession to maintain, they can afford to undercut, and underbid in fees, until they secure for themselves a patronage of some kind; and the public, or at least a large portion of the public, cannot understand how or why such a state of things exists. And the pretender is called a dentist, and looked upon by many as the equal of any other. To such an extent does this state of affairs exist, that it has become necessary for many competent ones to stoop to those low fees, to get a living for themselves and families.

Having pictured but a faint outline of the reality, we raise the question, is there a remedy? and what is it? Many things suggest themselves alike to the writer, and to those who read. But our main answer is, let there be the same effort that was applied to operative dentistry twenty years ago. At least, let every one give more attention to the artificial department, and elevate the standard beyond the ability of those into whose hands it has been allowed to come, and it will not be long until all can get a fair compensation for their services. The people will be better informed, and wholesale extraction, in a large measure, will be prevented. While we strive to elevate the department of artificial substitutes, let us maintain our present standard in operative dentistry.

We should be careful in the use of words, when in conversation with our patients or others, in reference to professional services. An improperly chosen word may convey the idea of a trade, or a mechanic in that line, when the proper word would imply skill, having back of it a knowledge of pathology and operative surgery. And in this connection I would refer, particularly, to the word *work*, as one very frequently used, if not improperly, it is certainly not used in good taste. The expressions in which this word is so frequently used will not inspire in the laity, that high-toned admiration for our profession that many of us so much desire.

How often do we hear such expressions as the following: "I do work for the family;" "I do all their work;" "He gets all his work done here;" "I have been doing their work;" "There can't anybody else work in his mouth;" "The work didn't give satisfaction;" "Brown used to do his work," etc., etc. Many more might be mentioned, but we forbear lest some one might think us to be as exacting and precise as the man who—

"Could a hair divide
"Twixt south and southwest side."

Very respectfully,

J. G. TEMPLETON.

